# The effects of arginine glutamate, a promising excipient for protein formulation, on cell viability: Comparisons with NaCl

**DOI:** 10.1016/j.tiv.2016.02.002

**Published:** 2016-06

**Authors:** Priscilla Kheddo, Alexander P. Golovanov, Kieran T. Mellody, Shahid Uddin, Christopher F. van der Walle, Rebecca J. Dearman

**Affiliations:** a Manchester Institute of Biotechnology, University of Manchester, Manchester M1 7DN, UK; b Faculty of Life Sciences, University of Manchester, Manchester M13 9PL, UK; c MedImmune Ltd, Granta Park, Cambridge CB21 6GH, UK

**Keywords:** 7-AAD, 7-aminoactinomycin D, ANOVA, one-way analysis of variance, APC, allophycocyanin, Arg·Glu, arginine glutamate, Arg·HCl, arginine hydrochloride, BSA, bovine serum albumin, DC, dendritic cell, EDTA, ethylenediaminetetraacetic acid, ELISA, enzyme-linked immunosorbant assay, FCS, fetal calf serum, FDA, Food and Drug Administration, FITC, fluorescein isothiocyanate, FSC-H, forward scatter, GRAS, Generally Recognized as Safe, HLA-DR, human leukocyte antigen, IC50, the concentration/osmolality required to cause a 50% loss in viability, ICAM-1, intercellular adhesion molecule 1, IL, interleukin, LPS, lipopolysaccharide, MFI, mean fluorescence intensity, NaGlu, sodium glutamate, PBS, phosphate buffered saline, PI, propidium iodide, SSC-H, side scatter, TLR, toll-like receptor, TNF, tumor necrosis factor, Arginine glutamate, Excipient, Monoclonal antibody, Apoptosis, Membrane markers, Inflammation

## Abstract

The effects of an equimolar mixture of l-arginine and l-glutamate (Arg·Glu) on cell viability and cellular stress using in vitro cell culture systems are examined with reference to NaCl, in the context of monoclonal antibody formulation. Cells relevant to subcutaneous administration were selected: the human monocyte cell line THP-1, grown as a single cell suspension, and adherent human primary fibroblasts. For THP-1 cells, the mechanism of cell death caused by relatively high salt concentrations was investigated and effects on cell activation/stress assessed as a function of changes in membrane marker and cytokine (interleukin-8) expression. These studies demonstrated that Arg·Glu does not have any further detrimental effects on THP-1 viability in comparison to NaCl at equivalent osmolalities, and that both salts at higher concentrations cause cell death by apoptosis; there was no significant effect on measures of THP-1 cellular stress/activation. For adherent fibroblasts, both salts caused significant toxicity at ~ 400 mOsm/kg, although Arg·Glu caused a more precipitous subsequent decline in viability than did NaCl. These data indicate that Arg·Glu is of equivalent toxicity to NaCl and that the mechanism of toxicity is such that cell death is unlikely to trigger inflammation upon subcutaneous injection in vivo.

## Highlights

• The effect of Arg·Glu on cell viability was examined with reference to NaCl.
• Arg·Glu is of equivalent toxicity to NaCl.
• The mechanism of cell death is via apoptosis and is thus not pro-inflammatory.
• Arg·Glu as a subcut excipient is not expected to result in atypical inflammation.

## 1 Introduction

Since the licencing by the US Food and Drug Administration (FDA) in 1986 of the first monoclonal antibody therapy, a murine IgG2a anti-CD3 antibody for the treatment of solid organ transplant rejection, the field has expanded rapidly (Hooks et al., 1991, Singleton, 2014, Walsh, 2010). These targeted biological therapies are now used for the treatment of a range of diseases including rheumatoid arthritis, psoriasis, inflammatory bowel disease and cancer (Lynch et al., 2014, Sedger and McDermott, 2014, Pandey and Mahadevan, 2014). Indeed, in 2012 three monoclonal antibodies that target the proinflammatory cytokine tumor necrosis factor (TNF)-α (adalimumab, infliximab and entanercept) were the world's top selling medicines in that year (Ho and Chien, 2012).

The formulation process in the biopharmaceutical industry requires the quantitative characterization of several potential degradation pathways, both covalent (e.g. oxidation, deamidation, proteolysis) and non-covalent (e.g. aggregation, unfolding-denaturation, phase separation) (Razinkov et al., 2015). The formulation that is selected must impart a long term stability to the product, at least over the required shelf-life, storage temperature and shipping conditions. It is the composition of the aqueous environment (buffer pH, ionic strength, excipient, etc.) that plays a critical role in maintaining the colloidal stability of an antibody in solution (Roberts et al., 2015). Various excipients (additives) have been used to optimize protein stability of pharmaceutical products and to minimize the extent of aggregation (Kamerzell et al., 2011). These include amino acids, such as histidine, arginine, and glycine (Arakawa et al., 2007a), sugars, such as trehalose (Kaushik and Bhat, 2003). There are several mechanisms by which small molecule excipients are proposed to stabilize proteins, including preferential interaction, preferential hydration and excluded volume (Bye et al., 2014). It is currently not possible to select excipients for a particular mAb formulation a priori, although platform approaches in the industry may favour one particular solution condition. The identification of one or more excipients to be included in a buffer in order to stabilize the protein formulation may be carried out by analysis of data generated by high throughput biophysical assays (Cheng et al., 2012).

Arginine is of particular interest as an excipient used to suppress protein aggregation, with several groups working on the elucidation of its mechanism of action. The weak interactions of arginine with the protein surface are due to its affinity for some amino acid side chains and the peptide backbone, but this is balanced by repulsion from the protein surface on account of an increase in the surface tension and volume exclusion effects (a complete review is given by Arakawa et al. (2007b)). The net effect of the preferential inclusion versus exclusion of arginine from the protein surface is dependent on its concentration, with an apparent saturation of the protein surface at around 0.5 M, above which arginine is considered to be excluded (Schneider and Trout, 2009). In silico modelling suggests that arginine cations cluster at certain protein surface patches via interaction with guanidinium and carboxylate amino acid side chains (Vagenende et al., 2013). However, the salt forms of excipients containing charged groups (or ion–ion pairs) are also known to play an important role in determining the mechanism of action in regard to protein stabilization. This is especially true for various salt forms of arginine, whose ability to suppress protein aggregation was shown to follow the empirical Hofmeister series; phosphate, sulphate and citrate more strongly interacting with the arginine cation and suppressing aggregation (Schneider et al., 2011). Given the importance of the salt form of arginine, one such excipient currently being explored in the area of biopharmaceutics is an equimolar mixture of amino acids l-arginine and l-glutamate (Arg·Glu salt). This excipient has been shown to increase protein solubility and stability and has been used in the fields of structural biology and vaccine development for some time (Blobel et al., 2011, Golovanov et al., 2004, Mistilis et al., 2015, Vedadi et al., 2006). These amino acids are a natural constituent of organisms and cells, they are part of human diet, and therefore inherently are non-toxic. Initial experiments have indicated that Arg·Glu can suppress aggregation of monoclonal antibody preparations induced by increased temperatures or pH and was also effective under accelerated stability conditions at weakly acidic to neutral pH (Kheddo et al., 2014).

In practice, excipients used for patient-injected formulations, including l-Arg and l-Glu, are chosen from the Generally Recognized as Safe (GRAS) category (US Food and Drugs Agency [FDA], 1982; Ogaji et al., 2011, Pifferi and Restani, 2003). However, the higher Arg·Glu concentrations (e.g. ≥ 200 mM in the final formulation, Kheddo et al., 2014), which may be necessary for optimal protein solubility, increase osmolality and hence may affect cell viability in vitro or in vivo or cause cellular stress (Vázquez-Rey and Lang, 2011). Indeed, the tolerability of hypertonic injectables has been reviewed recently (Wang, 2015). For drug products intended for subcutaneous injection, the main potential adverse effects were identified as enhanced site pain, local irritation and possible tissue damage and it was recommended that for drug products intended for subcutaneous injection, the upper osmolality limit should be 600 mOsm/kg (Wang, 2015). Changes to tissue osmolality may also cause activation of local dendritic cells (DC), sentinel cells of the immune system, acting as a trigger for inflammation (Gallo and Gallucci, 2013). These cells are activated by so-called “danger signals” which may derive directly from pathogens or dying host cells and also may be associated with perturbations of tissue/cell homeostasis such as changes in osmolality. Hypotonicity has been shown to act as a danger signal (Compan et al., 2012) and there are also reports that osmotic shock due to hypertonicity induced the production of the proinflammatory cytokine interleukin (IL)-8 by human peripheral blood mononuclear cells (Shapiro and Dinarello, 1997) and upregulation of macrophage caspase-1 (Ip and Medzhitov, 2015).

The aim of the current investigations was to examine the effects of increased concentrations of Arg·Glu on cell viability and cellular stress using in vitro cell culture systems. Similar studies have been conducted previously to investigate the impact of excipients within formulations on cell viability (Gursoy et al., 2003, Lee et al., 2013, Ménard et al., 2012, Nogueira et al., 2011). Cells of relevance to the subcutaneous route of administration have been selected (Kagan, 2014): the human monocyte cell line THP-1 (a surrogate DC line; Megherbi et al., 2009), grown as a single cell suspension, and human primary fibroblasts, cultured as an adherent monolayer. Thus, the impact on cell viability of increasing osmolalities of l-Arg and l-Glu solutions (together and separately) has been examined in comparison with the reference standard NaCl. In addition, for THP-1 cells, the mechanism of cell death which these different salts cause at relatively high concentrations has been investigated by flow cytometry, allowing differentiation between death by necrosis and death by apoptosis (otherwise known as programmed cell death) (Kabakov et al., 2011). More subtle effects of the presence of Arg·Glu on cell activation have been assessed as a function of changes in membrane marker expression on activated THP-1 cells (Megherbi et al., 2009).

## 2 Methods

### 2.1 Cell line maintenance

The THP-1 human monocytic leukaemia cell line (Sigma-Aldrich Chemical Co.; Poole, Dorset, U.K.) was cultured in RPMI-1640 medium (Sigma) supplemented with 400 μg/mL streptomycin, 400 μg/mL penicillin (both from Sigma), 2 mM l-glutamine (GIBCO; Paisley, Renfrewshire, UK) and 10% fetal calf serum (FCS; GE Healthcare, Cambridge, UK). THP-1 cells were maintained in vented T75 flasks at 37 °C in an atmosphere of 5% CO_2_ and split every 3–4 days when confluent (> 2 × 10^6^ cells/mL). Primary fibroblasts from human explants were cultured in DMEM medium (high glucose [4.5 g/L] with 2 mM l-Glutamine; GIBCO) supplemented with 400 μg/mL streptomycin, 400 μg/mL penicillin, 2 mM GlutaMax (GIBCO), 0.25 μg/mL amphotericin B (Sigma) and 10% FCS. Fibroblasts were maintained in 10 cm culture dishes at 37 °C/5% CO_2_ and passaged every 3–4 days when > 80% confluent. Cell number was assessed by exclusion of 0.5% trypan blue using a hemocytometer.

### 2.2 Salts used for generating changes in osmolality

Stock solutions of cell culture grade arginine glutamate (Arg·Glu) from equimolar mixtures of l-Arg (CAS number 74–79-3) and l-Glu (CAS number 142–47-2), NaCl (CAS number 7647–14-5), l-arginine hydrochloride (Arg·HCl; CAS number 1119–34-2) and sodium glutamate (NaGlu; CAS number 56–86-0) (all from Sigma-Aldrich) were prepared at 2.24 M, 4.96 M, 4.32 M and 3.9 M, respectively, in RPMI-1640 medium supplemented as described above without FCS. The salts of the amino acids (rather than the free bases) were used to keep the pH of l-Arg and l-Glu amino acid solutions within physiological range, whereas to prepare Arg·Glu, free bases of these amino acids were mixed together. Solutions were filtered using a 0.22 μm syringe filter and stored at 4 °C until use.

### 2.3 Determination of osmolality of salt solutions

The osmolality of Arg·Glu, NaCl, Arg·HCl and NaGlu solutions was measured using an Osmomat 030-D Cryoscopic Osmometer (Gonotec GmbH, Berlin, Germany) following standard operating procedures. A 1 M stock solution formulated in RPMI media was prepared for each salt and final concentrations of 5, 100, 150 and 200 mM were prepared for each compound for analysis and construction of a standard curve.

### 2.4 Cell treatments

Confluent THP-1 cells were harvested by centrifugation at room temperature (RT) (1000 *g* for 5 min) and re-suspended at 1 × 10^6^ cells/mL in RPMI-1640 medium without FCS in flat-bottomed 24 well tissue culture plates. Salts were prepared in the same medium at stock concentrations and added to cell cultures to achieve the required osmolalities (280–680 mOsm/kg). Control cells were treated with medium alone. In initial experiments, dose responses were conducted. In subsequent experiments, cells were treated with Arg·Glu, NaCl, Arg·HCl or NaGlu to achieve the osmolality range (280–680 mOsm/kg) or the equivalent concentration range 50–200 mM. In some experiments, positive control cells were treated with 0.1 μg/mL lipopolysaccharide (LPS) from *Escherichia coli* 055:B5 (Sigma). Cells were incubated for 4 h or for 24 h at 37 °C in an atmosphere of 5% CO_2_. Following the incubation, the cells were spun at 1000 *g* at RT for 5 min and re-suspended in 100 μL phosphate buffered saline (PBS; Sigma) without calcium and magnesium salts, for determination of cell viability. For phenotypic marker expression the cells were re-suspended in 2% bovine serum albumin (BSA; Sigma) in PBS. Supernatants and lysates were also harvested for nitric oxide determination. Lysates were obtained by lyzing the cell pellets in 100 μl of 0.01% Triton X 100 (Sigma).

Confluent fibroblast cells were washed once with PBS and trypsinized with 0.05% trypsin–ethylenediaminetetraacetic acid (EDTA; Sigma) for 3–4 min at 37 °C until the cells detached from the plate. Cells were re-suspended in complete DMEM medium and were centrifuged at 1000 *g* RT for 5 min. Cells were re-suspended at 2 × 10^5^ cells/mL in complete DMEM medium in flat-bottomed 24 well tissue culture plates for 6 h at 37 °C/5% CO_2_. The cells were then washed with PBS and treated with the salts formulated as described above but in DMEM medium without FCS to achieve the required osmolalities for 24 h. Following the incubation, the cells were trypsinized with 0.05% trypsin–EDTA and re-suspended in 5% FCS/PBS to determine cell viability.

### 2.5 Measurement of viability

Cell viability of both fibroblasts and THP-1 cells was routinely determined by staining of cells with 5 μg/mL propidium iodide (PI) immediately prior to analysis. Cells (10^4^) were analyzed using a FACSCalibur flow cytometer (Becton Dickinson, Mountain View, CA) and FlowJo software (Tree Star Inc., Ashland, OR, USA). Dose response curves were obtained and IC50 values (the concentration/osmolality required to cause a 50% loss in viability) calculated using the inbuilt dose–response fitting function with a nonlinear fit analysis in the OriginPro software version 9.0.

### 2.6 Measurement of phenotypic marker expression by flow cytometry

Following treatment of THP-1 cells, phenotypic marker expression was assessed. Cells were re-suspended in 2% BSA in PBS. Approximately 2 × 10^5^ cells were transferred to individual wells in round bottomed 96 well tissue culture plates and incubated at 4 °C for 15 min. The cells were washed at 1000 *g* for 5 min and incubated with the following monoclonal antibodies at 4 °C for 30 min: anti-human leukocyte antigen antibody (HLA-DR; DAKO, Glostrup, Denmark), anti-human CD54 antibody and allophycocyanin (APC)-conjugated anti-human CD86 antibody (BD PharMingen, Oxford, UK) at a 1 in 50 dilution. Isotype controls used were mouse IgG2aκ for anti-human HLA-DR and IgG1κ (BD PharMingen) for anti-human CD54 antibody and anti-human CD86 antibody. After incubation, cells were washed twice with PBS (1000 *g* for 5 min) followed by a further 30 min incubation at 4 °C with fluorescein isothiocyanate (FITC)-conjugated F(abʹ)_2_ goat anti-mouse IgG at a 1 in 50 dilution (DAKO) for anti-human CD54 and anti-human HLA-DR antibody stained samples; cells stained with APC-conjugated anti-human CD86 antibody were incubated with 2% BSA in PBS. Cells were washed as previously described and finally re-suspended in 5% FCS/PBS, and analyzed by FACSCalibur. Dead cells were excluded from all analyses by staining with 5 μg/mL PI immediately prior to analysis for cells stained for CD54 and HLA-DR; for CD86 staining dead cells were excluded following 5 min incubation with 2 μg/mL of 7-aminoactinomycin D (7-AAD; BD PharMingen). For each sample, a total of 10^4^ viable cells was analyzed.

Flow cytometry data were analyzed using FlowJo v10. Cell debris was eliminated by gating on the forward scatter (FSC-H) and side scatter (SSC-H) parameters and gates for marker expression were defined on the basis of isotype control staining. The mean fluorescence intensity (MFI) and the percentage positive cells were both used as separate indicators of the extent of surface marker expression.

### 2.7 Flow cytometric analyses for apoptosis and cytotoxicity

The levels of apoptosis/necrosis induced were investigated by staining of THP-1 cells for annexin V (TACS™ Annexin V-FITC Apoptosis Detection kits, R&D Systems Europe, Abingdon, UK). Following 4 h or 24 h incubation at 37 °C, the cells were incubated with PI and annexin V-FITC as described in the manufacturer's protocol and 10^4^ cells were analyzed by FACSCalibur and FlowJo. The combination of annexin V-FITC and PI staining allows for the differentiation between early apoptotic cells (annexin V^+^/PI^−^), late apoptotic cells (annexin V^+^/PI^+^), necrotic cells (annexin V^−^/PI^+^) and viable cells (annexin V^−^/PI^−^) (Dearman et al., 2008, Kabakov et al., 2011).

### 2.8 Analysis of nitric oxide release by Griess assay

The nitrite concentration from supernatants and lysates harvested at 24 h after initiation of THP-1 cell culture was measured by the Griess assay (Tsikas, 2007) according to the manufacturer's instructions (Promega, Southampton). A 6-fold dilution series using the nitrite standard (1.56 to 100 μM) was prepared and added to Maxisorb plastic microtiter plates (Nunc, Copenhagen, Denmark). Supernatants and lysate samples were added to the plate and 1% sulfanilamide in 5% phosphoric acid was added to every well. This was incubated for 10 min in the dark. N-1-napthylethylenediamine dihydrochloride (0.1%) in milliQ water was then added to every well and incubated for a further 10 min in the dark. Optical density at 550 nm was measured using an automated reader (Multiskan, Flow Laboratories, Irvine, Ayrshire, UK). The nitrite concentrations were determined by plotting the nitrite standard reference curve and using the associated computer software for microplate-based assays (Genesis, Life Sciences International Ltd., Basingstoke, UK).

### 2.9 Analysis of IL-8 secretion by enzyme-linked immunosorbant assay (ELISA)

The IL-8 content of culture supernatants harvested 24 h after initiation of culture of THP-1 cells was measured by sandwich ELISA (Duoset mouse IL-8 kit; R & D Systems, Abingdon, UK). Maxisorb plastic microtiter plates (Nunc, Copenhagen, Denmark) were used for these assays. Briefly, 96-well plates were coated with 0.1 μg/ml mouse anti-human IL-8 antibody and incubated overnight at 4 °C. 1% BSA in PBS was added to all wells to prevent nonspecific binding and the plates were placed on an orbital shaker (300 rpm) for 1 h at room temperature. Doubling dilutions of recombinant human IL-8 standard (0.008 to 2 ng/mL) were added to triplicate wells and supernatant samples diluted to various extents were added in duplicate and plates were incubated for a further 2 h with shaking. Goat anti-human IL-8 antibody (diluted 1 in 5000) was added to each well and the plates were incubated for 2 h. Streptavidin–horseradish peroxidase (diluted 1 in 1000) was added to each well and the plates incubated for 30 min in the dark. Between each step, the plates were washed three times with 0.05% PBS/Tween-20. Optical density at 450 nm was measured using an automated reader (Multiskan). A standard curve derived with murine recombinant cytokine and associated computer software for microplate-based assays (Genesis) were used to calculate the cytokine concentration in supernatants.

### 2.10 Statistical analyses

The statistical significance of differences in chemical or LPS induced changes in membrane marker expression, cytokine secretion and cell viability between experimental and control groups were calculated using one-way analysis of variance (ANOVA) with a P-value of < 0.05 being considered significant.

## 3 Results

### 3.1 Effect of Arg·Glu on THP-1 and primary fibroblast cell viability

**Fig. 1:**
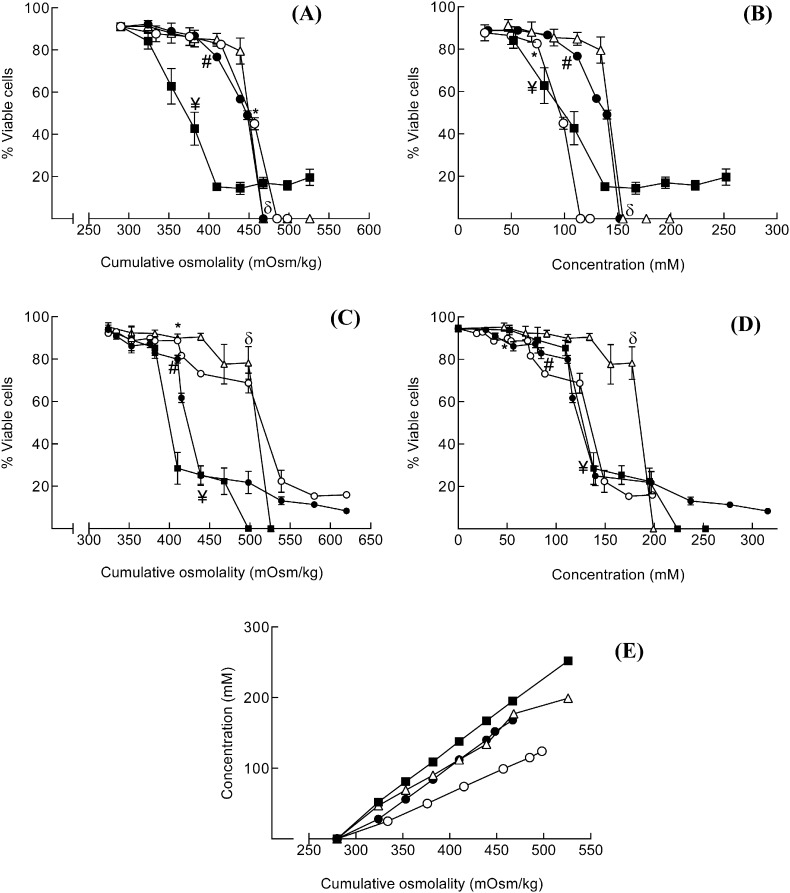
Effect of changes in osmolality on THP-1 cell or fibroblast viability: comparisons of different salts THP-1 cells were seeded into 24-well plates at 10^6^ cells/mL in serum free RPMI media (A, B) or human primary fibroblasts were seeded at 2 × 10^5^ cells/mL in complete DMEM medium in flat-bottomed 24 well plates for 6 h at 37 °C (C, D). Cells were cultured in serum free media for 24 h at 37 °C with varying concentrations of NaCl (○), Arg·Glu (●), Arg·HCl (■) and NaGlu (∆) spanning a range of osmolalities from 280 to 525 mOsm/kg (A) or from 280 to 625 mOsm/kg (C) and a range of concentrations from 0 to 250 mM (B) or from 0 to 325 mM (D). Control cells were cultured with medium alone (280 mOsm/kg). Following culture, fibroblasts were trypsinized and THP-1 and fibrobalsts were harvested and cell viability was determined using PI. Cells (10,000) were analyzed by flow cytometry using a FACSCalibur flow cytometer for PI staining (FL2 channel; viability) against forward scatter (FSC; size). Data are displayed as % cell viability for n = 3–6 experiments (mean and SE) versus excipient with respect to cumulative osmolality (A, C) and the concentrations of each excipient required to achieve the required osmolality (B, D). The statistical significance of differences between cells cultured in medium alone (isotonicity) and cells treated with various concentrations of salts was assessed by one way ANOVA (p < 0.05; * = NaCl, # = Arg·Glu, ¥ = Arg·HCl, δ = NaGlu). For clarity of presentation, only the first concentration of each salt preparation at which there was a significant loss of cell viability is illustrated. The relationship between each individual salt concentration as formulated in RPMI media and cumulative osmolality as measured using an osmometer is illustrated in (E).

In initial experiments, the ion-specific impact of changes in osmolality on viability of the THP-1 human monocyte cell line (grown as a cell suspension) was investigated. The excipients Arg·Glu, NaCl, Arg·HCl and NaGlu were used over a range of concentrations in order to achieve solution osmolalities in the range of 280–525 mOsm/kg (Fig. 1E). In these experiments the salts were added to the standard isotonic cell culture media, bringing the cumulative osmolality of solution into the hypertonic range. Cell viability was determined using flow cytometry (PI exclusion) and is illustrated as percentage viable cells for each treatment with respect to both solution osmolality (Fig. 1A) and solution concentration (Fig. 1B). Baseline viability (in the presence of media alone) for THP-1 cells was ~ 90%. Increasing the cumulative osmolality of the culture media with Arg·Glu, NaCl and NaGlu caused a decrease in cell viability, with toxicity profiles that were virtually superimposable with ~ 50% toxicity recorded at 450 mOsm/kg. However, treatment with Arg·HCl caused a more marked loss in cell viability at much lower cumulative osmolalities, with ~ 45% toxicity observed at 380 mOsm/kg. To facilitate direct comparison of the relative amount of each ion required to cause significant toxicity, the same cytotoxicity data are displayed also as a function of the concentrations of the individual salts added to the isotonic media (Fig. 1B). This comparison shows that on a mole per mole basis, the toxicity profiles of NaGlu and Arg·Glu are superimposable, whereas Arg·HCl and NaCl cause losses in cell viability at lower concentrations (~ 50% toxicity at 100 mM). In contrast, the impact on cell viability of addition of Arg·Glu at concentrations below 100 mM is minimal.

A somewhat different picture was seen when parallel experiments were conducted with adherent primary human fibroblasts. Fibroblast cells were cultured at 2 × 10^5^ cells/mL with a range of osmolalities (280–620 mOsm/kg) of the various salts for 24 h and the cell viability was determined by PI staining using flow cytometry; data are illustrated with respect to osmolality (Fig. 1C) and concentration (Fig. 1D). For all salts there was a dose dependent decrease in cell viability (reaching statistical significance between 385 and 425 mOsm/kg) and for some salts (Arg·Glu and NaCl) there was a residual population of ~ 20% that remained viable. With respect to osmolality changes, the cytotoxicity profiles of Arg·Glu and Arg·HCl showed a rapid decrease in viability from 400 mOsm/kg and above, which was very similar to the dose response observed for THP-1 cells. Fibroblasts were more resistant to the effects of NaCl and NaGlu, where a slower decline in viability was observed until ~ 525 mOsm/kg, which was followed by a very rapid drop in viability (Fig. 1C). However, on a molar concentration basis, all salts with the exception of NaGlu displayed equivalent toxicity over the range of concentrations examined (50–200 mM) (Fig. 1D). These data indicate that for the adherent fibroblast cell line, Arg·Glu and Arg·HCl have some effects on cell viability due to factors in addition to osmolality.

An alternative way of representing these data for comparative purposes is to calculate the concentration/osmolality that results in a 50% loss of viability, an IC50 value (Table 1) for each salt. For THP-1 cells, the IC50 values confirmed that with respect to osmolality, Arg·Glu, NaCl, and NaGlu had similar effects on viability (values ranging from 449.1 and 474.3 mOsm/kg) whereas the IC50 value for Arg·HCl was considerably lower (360.6 mOsm/kg). With respect to molar concentration, however, IC50 values for NaCl and Arg·HCl were considerably lower (< 100 mM) than those recorded for NaGlu and Arg·Glu. For fibroblast cells, IC50 values for osmolality confirmed that these cells were more susceptible to Arg·Glu and Arg·HCl than to NaCl and NaGlu. On a mole for mole basis, similar IC50 values (ranging from 119.1 to 128.0 mM) were recorded for all of the salts with the exception of NaGlu (171.1 mM).

Interestingly, as the independent osmolality measurements show (Fig. 1E), in practice the increase in osmolality versus concentration of salt added (Arg·Glu, NaCl, Arg·HCl and NaGlu) is highly dependent on the nature of the salt, likely due to differences in their degree of dissociation at equal concentrations. The NaCl salt results in the highest increase in osmolality of the solution, and the Arg·HCl salt the least, for each given concentration of salt. Thus, considerably higher concentrations of Arg·Glu, Arg·HCl and NaGlu are required to achieve osmolalities equivalent to NaCl (Fig. 1E).

In summary, the key findings here were that the effects on cell viability were largely consistent with changes in osmolality. However, for Arg·Glu and Arg·HCl with the adherent cells and for Arg·HCl with the THP-1 cells, the effects were not solely attributable to osmolality changes. In subsequent experiments, the focus was on THP-1 cells due to the ability of these cells to act as indicator cells and to respond to danger signals (Gallo and Gallucci, 2013, Megherbi et al., 2009).

### 3.2 Toxicity of l-Arg salts for THP-1 cells is not due to nitric oxide production

**Fig. 2:**
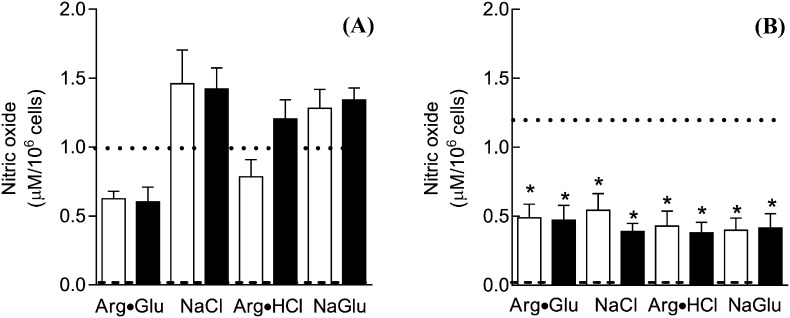
Impact of Arg·Glu and other salts on THP-1 cell nitric oxide production THP-1 cells were seeded into 24-well plates at 10^6^ cells/mL in serum free RPMI media and were cultured for 24 h at 37 °C in medium alone (represented by the dashed line) or in the presence of 50 mM (open column) or 100 mM (closed column) Arg·Glu, NaCl, Arg·HCl or NaGlu. Supernatants (A) and lysates (B) were collected after the 24 h incubation and the nitrite concentration was determined by the Griess assay. The nitric oxide content of the media (with supplements) in the absence of cells was also determined (dotted line). The nitrite concentrations were determined using a nitrite standard reference curve and the associated computer software for microplate-based assays and data are displayed as nitrite concentration (μM; mean and SE). The statistical significance of differences in the nitrite content of samples from cells cultured in medium alone and cells treated with various concentrations of salts was assessed by one way ANOVA (*p < 0.05).

A potential mechanism for the additional impact of hypertonic Arg·HCl on THP-1 cell viability, over and above the effects of changes in osmolality, was explored. It was hypothesized that the more profound cytotoxic effect of Arg·HCl could be due to the release of nitric oxide by the cells in the presence of Arg·HCl, as arginine is a substrate for the formation of nitric oxide (Chen et al., 2013, Chou et al., 2010, Pekarova et al., 2013). Therefore, the effects of the addition of Arg·Glu, NaCl, Arg·HCl and NaGlu on nitric oxide production by THP-1 cells were examined (Fig. 2). Cells were cultured for 24 h in the presence of 50 or 100 mM concentrations of the salts, the latter condition under which Arg·HCl caused marked toxicity, and supernatants and cell lysates analyzed for secreted and intracellular levels of nitric oxide, respectively, by Greiss assay. Although the nitric oxide content of supernatants derived from cells following treatment with the various salts was variable (~ 60% to 140% of control values of 1 μM), such differences were not statistically significant. In contrast, statistically significant decreases in the nitric oxide content of lysates (intracellular nitric oxide content) were recorded for all treatments (ranging from ~ 30% to 40% of control values of 1.2 μM). However, the divergent cytotoxicity profile recorded for Arg·HCl compared to the other salts could not be reconciled on the basis of nitric oxide production.

### 3.3 Mechanism of cell death caused by hypertonic addition of salts to THP-1 cells

**Fig. 3:**
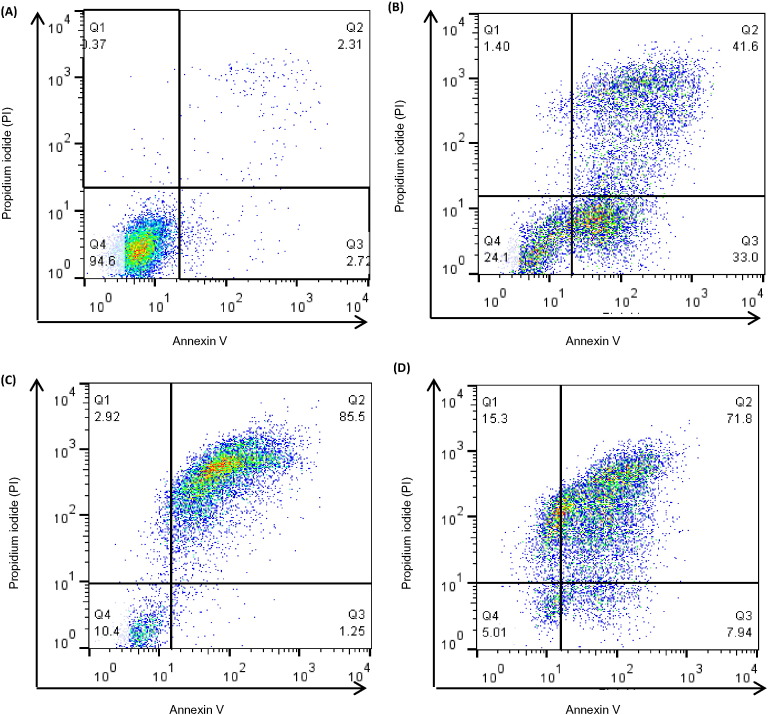
The gating strategy for the characterization of the mechanism of cell death (representative FACS histograms) THP-1 cells were seeded into 24-well plates at 10^6^ cells/mL and cultured for 24 h in serum free RPMI media alone (A), or in the presence of 150 mM NaGlu (B), 200 mM Arg·Glu (C), 200 mM NaCl (D). Following incubation, cells were stained with annexin V-FITC (FL-1 channel) and PI (FL-2 channel) and 10,000 cells were analyzed using a FACSCalibur flow cytometer. Data are displayed as representative quadrant analyses showing the pattern of cytotoxicity for each treatment group; in each case the lower left quadrant represents Annexin V-ve/PI-ve (viable) cells, the lower right quadrant represents annexin V + ve/PI-ve (early apoptotic) cells, the upper right quadrant represents annexin V + ve/PI + ve (late apoptotic) cells and the upper left quadrant represents annexin V-ve/PI + ve (necrotic) cells. The percentage of cells in each quadrant is indicated.

**Fig. 4:**
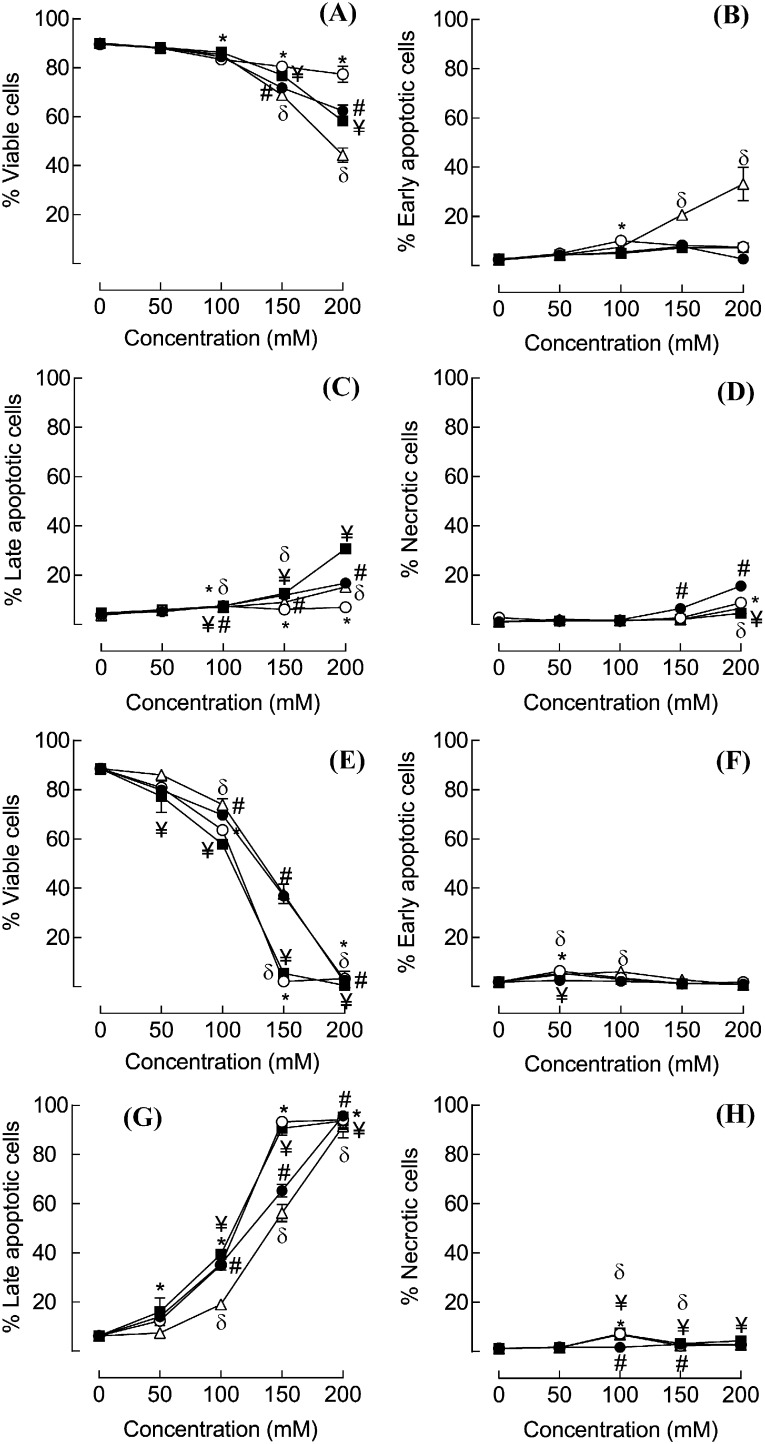
Characterization of the mechanism of cell death THP-1 cells were seeded into 24-well plates at 10^6^ cells/mL in serum free RPMI media and were cultured for 4 h (A–D) or 24 h (E–H) at 37 °C in the presence of 50–200 mM NaCl (○), Arg·Glu (●), Arg·HCl (■) or NaGlu (∆). Control cells were cultured with medium alone. To characterize the extent and pattern of induced cytotoxicity, cells were stained with Annexin V-FITC (FL-1 channel) and PI (FL-2 channel) and 10,000 cells were analyzed using a FACSCalibur flow cytometer. The results are displayed as the percentages of cells that are Annexin V-ve/PI-ve (viable) (A, E), Annexin V + ve/PI-ve (early apoptotic) (B, F), Annexin V + ve/PI + ve (late apoptotic) (C, G), Annexin V-ve/PI + ve (necrotic) (D, H). In each case, data are shown as % total cells in each category (mean and SE for n = 3 experiments, where for clarity SE > 2% only are shown). The statistical significance of differences between cells cultured in medium alone and cells treated with various concentrations of salts was assessed by one way ANOVA (p < 0.05; * = NaCl, # = Arg·Glu, ¥ = Arg·HCl, δ = NaGlu).

In initial experiments (cf Fig. 1) cell viability was assessed using PI staining which distinguishes dead (necrotic) cells from live cells. In those experiments, Arg·Glu and NaGlu showed very similar toxicity profiles with respect to solution osmolality and cell death to that which was observed for the reference excipient, NaCl. However, to further characterize the extent and pattern of cell death occurring, and to confirm that the mechanism of cell death was indeed similar between Arg·Glu and NaCl, a combination of PI and annexin V-FITC staining was used. This combination allows for the distinction between early apoptotic cells (annexin V-FITC positive), late apoptotic cells (annexin V-FITC and PI positive), necrotic cells (PI positive) and viable cells (unstained) (Kabakov et al., 2011). THP-1 cells were treated with 50–200 mM concentrations of the salts, or with medium alone, for 4 h or 24 h, stained with annexin V-FITC and PI and analyzed using a FACSCalibur flow cytometer (Fig. 3, Fig. 4). These time points were chosen on the basis of preliminary kinetics experiments that revealed that after 2 h incubation there was little impact on THP-1 cell viability or apoptosis, whereas after 4 h, significant effects were observed at concentrations of > 150 mM (data not shown). The 24 h time point was utilized to determine whether lower concentrations (< 100 mM) of salts that were without effect on cell viability as measured by PI exclusion at this time point (cf Fig. 1) had more subtle effects on apoptosis. Representative quadrant plots are displayed in Fig. 3, illustrating the gating strategy and showing examples of predominantly viable cells (Fig. 3A, 94.5% cells in lower left quadrant), populations of early and late apoptotic cells (Fig. 3B; 33% and 42%, respectively, in the lower and upper right quadrants), predominantly late apoptotic cells (Fig. 3C; 85.5% of cells in upper right quadrant) and populations containing necrotic cells (Fig. 3D; 15.5% cells in the upper left quadrant).

Culture of THP-1 cells with 50 or 100 mM of the salts for 4 h had little impact on cell viability (Fig. 4A-D), although there was a significant loss in viability for all 4 salts at concentrations of 150 mM and above. This was generally paralleled by increased numbers of late apoptotic and necrotic cells, with the exception of NaGlu, which caused a more marked loss in viability than did the other salts, and this was paralleled by an increased frequency of early apoptotic cells (Fig. 4B). After 24 h in culture, only the 50 mM concentration of each of the salts was without significant effects on cell viability. In each case at doses of 100 mM and above there was a significant drop in the frequency of viable cells which was reflected by a corresponding significant increase in late apoptotic cells, with no marked changes in early apoptotic or necrotic cells (Fig. 4E–H). Consistent with the previous data (cf Fig. 1B) where viability was assessed simply as a function of PI exclusion, for a given concentration of salt, NaCl and Arg·HCl were considerably more cytotoxic than NaGlu or Arg·Glu and this resulted in increased numbers of late apoptotic cells.

These data demonstrate that the mechanism of cell death is very similar between NaCl and Arg·Glu, with loss of viability being paralleled in both cases by increased frequency of late apoptotic cells with little evidence of early apoptotic or necrotic cells.

### 3.4 Impact of hypertonic concentrations of salts on markers of THP-1 cell activation

**Fig. 5:**
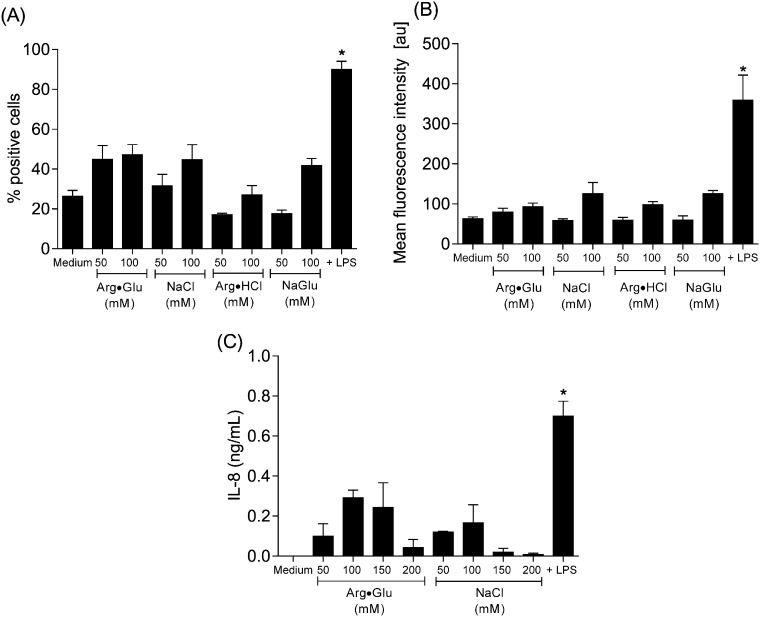
Effects of Arg·Glu and other salts on CD54 expression and IL-8 production THP-1 cells were seeded into 24-well plates at 10^6^ cells/mL in serum free RPMI media and were cultured for 24 h at 37 °C with medium alone or in the presence of 50–200 mM of Arg·Glu, NaCl, Arg·HCl or NaGlu. Positive control cells were cultured with LPS (0.1 μg/mL). At the end of the culture period, cells were stained for expression of CD54 or with isotype control (mouse IgG1κ) antibody and dead cells were excluded with PI. Data (10,000 cells) were acquired using a FACSCalibur flow cytometer and results are displayed as percentage positive cells (A) and mean fluorescence intensity (arbitrary units; au) (B) (mean and SE for six independent experiments). Following culture, samples were centrifuged at 1000 rpm for 5 min and supernatants were collected and analyzed for IL-8 content by ELISA (C). Results are displayed as mean and SEM of 3 independent experiments. The statistical significance of differences between cells cultured in medium alone and cells treated with various concentrations of salts or LPS was assessed by one way ANOVA (*p < 0.05).

It is clear from the experiments described thus far that high concentrations (> 150 mM) of Arg·Glu and other ions impact markedly on cell viability over a 24 h culture period. In subsequent experiments, more subtle effects on the activation status of the cells were explored under conditions where the impact on cell viability was marginal. Changes in membrane marker (CD54, HLA-DR and CD86) expression were measured on resting THP-1 cells and cells stimulated with the toll-like receptor (TLR) 4 ligand LPS, a polyclonal activator of monocytes (Rossol et al., 2011). Resting THP-1 cells were ~ 20% CD54 + ve, 60% HLA-DR + ve and < 5% CD86 + ve. Treatment with LPS increased significantly the frequency of CD54 + ve cells (to ~ 90%) and also upregulated the level of expression (increasing MFI from ~ 60 au to 360 au) but was without marked effect on levels of the other markers. The presence of the various salts was also without impact on resting levels of HLA-DR and CD86 (data not shown). With respect to CD54 expression, 50 mM of the salts were largely without effect on either the frequency of positive cells or the extent of expression, whereas 100 mM of each of the salts tended to cause a small increase (to ~ 40%) in the proportion of CD54 + cells and a slight increase in MFI to ~ 120 au for NaCl. However, none of these small changes reached statistical significance (Fig. 5A and B). Finally, the impact of Arg·Glu and NaCl on production of the proinflammatory cytokine IL-8 was examined. Cells cultured with medium alone did not secrete detectable levels of IL-8 (< 0.1 ng/mL) whereas culture in the presence of LPS induced detectable cytokine expression (~ 1 ng/mL) (Fig. 5C). There was some inter-experimental variation, but treatment with a range of concentrations of Arg·Glu or NaCl did not result in significant cytokine production.

## 4 Discussion

The impact of the excipient Arg·Glu and other related salts on cell viability and cellular stress using in vitro cell culture systems has been examined. These studies have demonstrated that Arg·Glu does not have any further detrimental effects on viability of the monocyte (DC surrogate) THP-1 cell line in comparison to NaCl at equivalent osmolalities, and that relatively high concentrations of both salts cause cell death by apoptosis. In contrast, Arg·HCl was more toxic to this cell line at equivalent osmolalities. For human primary fibroblasts that grow as an adherent monolayer, all salts caused significant toxicity at 385 and 425 mOsm/kg, although Arg·Glu and Arg·HCl caused a more precipitous subsequent decline in viability than did NaCl or NaGlu. These data suggest that salts containing arginine have an impact on cell viability of adherent cells that is in addition to effects due to hypertonicity and that in the case of Arg·Glu can only be partially ameliorated by the presence of Glu. There was some indication that the arginine containing salts affected cell adherence/morphology of the primary human fibroblasts (data not shown). Indeed there is some precedent for hypertonicity impacting on the cytoskeleton, with adherent human monocytes and macrophages showing changes in microtubule organisation upon hypertonic stress (400 mOsm/kg NaCl) (Nunes et al., 2013). It is of interest that the polystyrene tissue culture plates in which the primary human fibroblasts are cultured are treated to increase hydrophilicity of the polystyrene and ensure a negative charge, conditions which promote cell adherence (Curtis et al., 1983). It may be that the positively charged arginine ion in the Arg·Glu and Arg·HCl preparations interfere with this interaction through salt screening of the net surface charge.

It is of interest that in the nonadherent indicator cell line (THP-1), Arg·HCl was more toxic than the other salts, for a given osmolality. For the nonadherent cell line, the presence of Glu in the Arg·Glu preparation reduced this toxicity. One possible explanation for this increased toxicity was that arginine was acting as a substrate for the formation of nitric oxide (Chen et al., 2013, Chou et al., 2010, Pekarova et al., 2013), which interacts with superoxide radicals to form peroxynitrite, causing oxidative damage to cells (Calcerrada et al., 2011). Clearly the THP-1 cells were able to secrete detectable nitric oxide (~ 1 μM was measured in the supernatants, levels some 50-fold higher than that recorded in the absence of cells), amounts similar to those recorded previously for the same cell line under the same culture conditions (~ 2.5 M) (Garg et al., 2005). Interestingly, there was no differential effect of arginine on nitric oxide production. However, all salts caused a significant decrease in intracellular nitric oxide, even under conditions (50 mM) where there was no effect on viability, suggesting that this may be due to hypertonicity. Indeed, it has been reported previously in other cell types (muscle cells and in cartilage) that exposure to hypertonic concentrations of NaCl and sucrose or mannitol results in reduced levels of nitric oxide and the enzyme inducible nitric oxide synthase (Le et al., 2006, Pingle et al., 2003). Thus, hypertonicity per se may trigger decreases in expression of nitric oxide. It should be noted that the assay utilized herein is an approximation of nitric oxide content, as it measures nitrite (NO^2 −^) only, which is one of two primary, stable and nonvolatile breakdown products of nitric oxide, the other being nitrate (NO^3 −^)(Tsikas, 2007). The mechanism by which Arg·HCl is more toxic for the nonadherent cell line than the other salts is unclear at present, however, the presence of Glu in the Arg·Glu mixture appears to ameliorate this toxicity.

The experiments conducted herein have demonstrated that hypertonic stress induced by all 4 salts tested resulted in cell death by apoptosis (with the majority of cells in the late apoptotic stage by 24 h) with very little necrosis occurring at any concentration. This is a key finding given the fact that cells that die by necrosis expel their contents when they lyse, including inflammatory cytokines that stimulate further pro-inflammatory responses (Rock and Kono, 2008). In contrast, cells that die by apoptosis are compacted into apoptotic bodies as a mechanism to avoid immune activation and instead are phagocytosed (Biermann et al., 2013). One of the earliest signs of apoptosis is the translocation of the membrane phospholipid phosphatidylserine from the inner to the outer leaflet of the plasma membrane. This molecule is recognized by phagocytes, triggering phagocytosis and also the production of anti-inflammatory cytokines, resulting in efficient removal of the dying cell without inflammation. In the flow cytometric analyses of apoptosis, it is the expression of this cell surface phosphatidylserine that is detected by the binding of the fluorescently labelled ligand annexin V (Kabakov et al., 2011). Although there have been suggestions that late apoptotic cells more closely resemble necrotic cells, due to their compromised cell membranes, in fact it has been shown that both early and late apoptotic cells induce the differential signalling pathways in macrophages compared with necrotic cells and that intracellular contents released from late apoptotic cells are neutral and not proinflammatory (Patel et al., 2006). The fact that all of the salts examined caused death by apoptosis is not simply a feature of the target THP-1 cell, as it has been demonstrated previously that under the right conditions, such as stimulation with bacterial toxins, THP-1 cells do undergo necrosis (Melehani et al., 2015). It is of interest that all of the salts caused cell death by the same mechanism and with similar kinetics. This suggests that induction of apoptosis is a common feature of hypertonic insult which is consistent with previous studies in which hyperosmotic stress has been shown to induce apoptosis, particularly in kidney cells (Alfieri and Petronini, 2007). There are fewer studies in which monocyte or DC populations have been investigated, but here too the mechanism of monocyte cell death induced by osmolytes such as NaCl and mannitol at 500 mOsm/kg has been shown to be apoptosis (Gastaldello et al., 2008). To our knowledge there have been no previous studies on the mechanism of Arg·Glu induced cytotoxicity. Thus, all the salts including Arg·Glu induce the THP-1 cells to undergo apoptosis, in which case this is not expected to result in inflammation.

More subtle effects of the salts on the THP-1 indicator cell line were explored: the impact on markers of cellular stress/cell maturation such as changes in expression of the membrane marker CD54 (intercellular adhesion molecule-1; ICAM-1) and production of the pro-inflammatory cytokine IL-8. ICAM-1 is an adhesion molecule that is a member of the immunoglobulin superfamily with functions that relate to its role in cell adhesion and migration (Simmons, 1995). Its ligands include the matrix factor hyaluronan and lymphocyte function-associated antigen-1 on T cells; interaction with its T cell ligand provides costimulation and therefore activation of the adaptive immune response (Simon et al., 1991). IL-8 is an inducible chemokine whose production is stimulated in various cell types by other proinflammatory cytokines such as IL-1 and TNF-α. IL-8 plays a key role in acute inflammation, being the main chemotactic factor for neutrophils, the most important phagocytic cell in many different types of inflammatory reaction and may also play a role in macrophage and fibroblast chemotaxis (Harada et al., 1994). These two markers have been shown to be indicative of the activation of DC-like cells such as the THP-1 cell line. Thus, TLR ligands including TLR1, TLR2 and TLR4 each caused increased ICAM-1 expression and IL-8 secretion by THP-1 cells (Lee et al., 2011, Spachidou et al., 2007). Furthermore, less vigorous stimuli, such as chemicals that have the ability to cause contact allergy, a T cell mediated delayed type hypersensitivity in the skin, upregulate the same markers on THP-1 cells in culture (Megherbi et al., 2009, Tuschl and Kovac, 2001). In the experiments described herein, consistent with the published data, stimulation of THP-1 cells with the TLR4 ligand LPS resulted in the expected up-regulation of these markers, with increases in both the number of ICAM-1 positive cells and the amount expressed per cell. In contrast, treatment with the salts was without marked effects on expression of the membrane marker or cytokine expression; for both Arg·Glu and NaCl there were similar, but variable, low level effects on both markers that did not reach statistical significance. There is some evidence with other cell lines that hyperosmolality may impact on cytokine expression, particularly for endothelial and epithelial cell lines. Thus, hypertonic concentrations of NaCl and mannitol increased IL-8 expression by Caco-2 and HT-29 cells (human intestinal epithelial cell lines) and by bronchial epithelial cells (NCl-H_292_) (Hashimoto et al., 1999, Hubert et al., 2004, Németh et al., 2002). In both cell types, induction of IL-8 was via a p38 mitogen-activated protein kinase. Interestingly, for the human intestinal epithelial cell lines, hypo-osmolality (163 mOsm/L NaCl) was considerably more stimulatory than was hyperosmolality (496 mOsm/L NaCl) with respect to cytokine induction (Hubert et al., 2004). It could be argued that for tissues such as the gastrointestinal tract, exposure to marked fluctuations in osmolarity is a feature of normal physiology and as such these cells are required to sense and respond to such changes (Brocker et al., 2012). In contrast, there are far fewer reports regarding the response of monocytes/macrophages or DC to hyperosmotic stress (induced by either NaCl or glycerol/mannitol), and conflicting results have been generated. It has been reported that hypertonicity had no impact on basal IL-8 release and inhibited LPS-induced expression of this cytokine by freshly isolated human blood monocytes (Brulez et al., 1999), whereas others report that human peripheral blood mononuclear cells respond to NaCl by upregulating IL-8 and IL-1β, the latter at the level of message only (Shapiro and Dinarello, 1997). Furthermore, it has been demonstrated that hypotonicity, but not hypertonicity, activates macrophage and monocyte caspase 1 (IL-1β processing enzyme) and induces IL-1β release as a result of cell swelling (Compan et al., 2012, Perregaux et al., 1996). Conflicting data have been reported, with hyper-osmotic stress upregulating caspase 1, but here macrophages were primed with LPS (Ip and Medzhitov, 2015). As such, the experiments reported herein confirm that in the absence of priming, hypertonicity caused by Arg·Glu or NaCl fails to impact significantly on monocyte IL-8 production, over a range of doses that spanned concentrations that impacted on cell viability. In regard to excipient concentrations, it should be remembered that immediately following subcutaneous injection the vehicle in which the drug is formulated disperses. This exchange into a physiological medium, with concomitant decrease in excipient concentration, may have biophysical consequences and the in vivo local cellular response is yet to be determined (Kinnunen and Mrsny, 2014).

In conclusion, adherent and nonadherent cell lines exhibit very similar toxicity profiles for Arg·Glu, and for the nonadherent cell line, the data are consistent with the toxicity being due to changes in osmolality. In common with hypertonic concentrations of NaCl, the mechanism of cell death is via apoptosis and is thus not pro-inflammatory, which is confirmed by the negative findings for markers of cellular stress or activation (membrane marker and proinflammatory cytokine expression). Taken together these data indicate that Arg·Glu is of equivalent toxicity to NaCl and that the use of Arg·Glu as an excipient for subcutaneous injection is not expected to result in atypical inflammation.

## Transparency document

Transparency document

## Figures and Tables

**Table 1:** IC50 values calculated additive concentrations and osmolalities required to achieve 50% drop in viability for THP-1 cells or fibroblasts for each of the salts; raw data displayed in Fig. 1.

Additives	THP-1 cells		Fibroblast	
	[Salt], mM	mOsm/kg	[Salt], mM	mOsm/kg
Arg·Glu	141.1	449.1	119.1	417.4
NaCl	99.6	456.4	114.7	482.9
Arg·HCl	94.3	360.6	128.0	400.7
NaGlu	158.6	474.3	171.1	489.7
